# Multiscale Modeling Analysis of the Mechanical Behaviors and Failures of In Situ Particle Reinforced Titanium Matrix Composites Based on Microstructural Characteristics

**DOI:** 10.3390/ma19010035

**Published:** 2025-12-21

**Authors:** Xixi Geng, Kejian Li, Zhiyang Liao, Zhipeng Li, Zhipeng Cai, Qu Liu

**Affiliations:** 1 Department of Mechanical Engineering, Tsinghua University, Beijing 100084, China; gqq23@mails.tsinghua.edu.cn (X.G.); kejianli@mail.tsinghua.edu.cn (K.L.); liao-zy18@outlook.com (Z.L.); czpdme@mail.tsinghua.edu.cn (Z.C.); 2 Avic Shenyang Aircraft Design & Research Institute, Shenyang 110035, China; 01482111@163.com

**Keywords:** particle reinforced titanium matrix composites, multiscale model, mechanical behaviors, damage evolution

## Abstract

A multiscale model is developed to investigate the mechanical behavior and failure of in situ particle reinforced titanium matrix composites (PTMCs). Through the microstructural observation of the heterogeneous microscopic and mesoscopic structures in the in situ TiB/Ti55531 composites, multiscale heterogeneous models coupled to the finite element method are employed to simulate the mechanical behaviors and failures. In the atomic scale, molecular dynamics (MD) simulations are applied to determine the traction-separation (T-S) responses of the cohesive zone model (CZM) describing the Ti/TiB interface. Then, the mesoscale representative volume element (RVE) model with heterogeneous structure, including the Ti55531 matrix, the TiB particles, and their interfaces represented by the parameterized CZM, is established. The volume fraction and distribution morphology of TiB particles result from the microstructural analysis of titanium matrix composites. The simulation results show that the Young’s modulus, tensile strength and elongation of multiscale are in excellent agreement with experimental results. The stress transfer, damage evolution and fracture behavior of the TiB particles in the composites are also analyzed using this multiscale approach.

## 1. Introduction

Titanium matrix composites (TMCs) have attracted extensive attention due to their high specific strength and modulus, excellent wear resistance and good high-temperature performance [1]. As a kind of discontinuously reinforced titanium matrix composites, PTMCs can be made by conventional casting or powder metallurgy methods at low cost. Moreover, PTMCs exhibit good isotropy [2] and excellent formability for subsequent processing [3]. Common ceramic reinforcements include SiC, Al_2_O_3_, TiC, TiB and TiB_2_. Among these, TiB is considered the best reinforcement because it has nearly the same density and thermal expansion as titanium [4,5], resulting in low residual stress during processing. Meanwhile, the TiB phase is thermodynamically stable in the titanium matrix, forming a clean and well-bonded interface [6].

Due to the heterogeneous microscale and mesoscale structures of PTMCs, it is expensive and challenging to investigate their mechanical properties only by experiment. It is also difficult to reveal the influence of microscale and mesoscale structures on the mechanical behavior of composites and the physical mechanisms of the damage evolution during fracture. Therefore, multiscale simulation is essential. Multiscale simulations can obtain interfacial parameters at the atomic scale, which are difficult to measure by experiment. Then, these parameters can be introduced into a mesoscale model to analyze the stress–strain distribution as well as the initiation and propagation of cracks between matrix and reinforcements. Moreover, the mechanical properties obtained from mesoscale simulations can be further introduced into higher-scale homogenized models, thus establishing a comprehensive link between microstructure and macroscale behavior of composites. This approach provides critical insights into their mechanical performance, fracture mechanisms and offers valuable guidance for material design and optimization.

Previous studies have demonstrated that multiscale modeling is an effective way to simulate interfacial behavior and mechanical response of PTMCs. Sazgar et al. [7] modeled the interfacial behavior of Al/Al_2_O_3_ composites and performed molecular dynamics (MD) simulations at different temperatures. The traction-separation (T-S) curves obtained from the tensile and shear simulations were fitted to obtain the constitutive parameters of a cohesive zone model (CZM). The CZM was subsequently implemented in a mesoscale two-dimensional representative volume element (RVE) model. The simulated tensile results showed excellent agreement with experimental data. By varying particle volume fractions, the mechanical properties of Al/Al_2_O_3_ metal matrix composites were also predicted with acceptable accuracy. It can then be replaced by a homogenized model, enabling more efficient simulations in the following studies. Elkhateeb et al. [8] constructed a microscale model for an α-TiAl/β-TiV/TiC three-phase interface in Ti6Al4V/TiC composites. And MD simulations were performed at different temperatures to develop a parameterized CZM. The CZM was introduced into the macroscale finite element simulations to verify the model’s validity. Similarly, Wang et al. [9] developed a SiC/pyrolytic carbon (PyC) interface model and carried out MD simulations. The interfacial parameters obtained were incorporated into a finite element model for uniaxial tensile simulations, and the results were consistent with experimental results.

At present, multiscale simulations focusing on in situ TiB reinforced titanium matrix composites (TMCs) still remain limited. Attarian et al. [10] studied TiB reinforced TMCs at the microscale and mesoscale, but their RVE model adopted several simplifications. For example, the particles not only have identical sizes, but also have the same orientations, and interfacial damage was not considered. In addition, no experimental investigation was performed. Liu et al. [11] conducted mesoscale simulations for TiB/IMI834 composites, although the interfacial properties were not investigated. In this study, an in situ TiB/Ti55531 (Ti-5Al-5Mo-5V-3Cr-1Zr) composite was investigated using a multiscale modeling framework. In the microscale, MD simulations were employed to obtain the interfacial mechanical parameters. These parameters were subsequently introduced into a mesoscale three-dimensional RVE model by CZM to simulate the tensile behavior of the composite. The reliability of the simulation results was verified by experiments. This work provides a foundation for further studies on the mechanisms influencing the mechanical properties, the damage evolution of the material and the development of homogenized models at larger scale.

## 2. Materials and Experiments

### 2.1. Metallographic Observation

**Figure 1:**
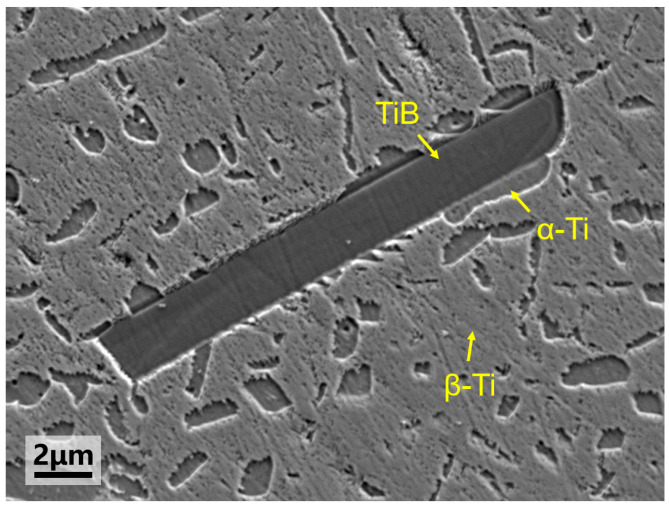
Metallographic SEM image of the TiB/Ti55531 composite.

The metallographic specimen of the TiB/Ti55531 composite was observed by scanning electron microscopy (SEM), as shown in Figure 1. The SEM observations were performed using a GeminiSEM300 (Carl Zeiss Microscopy GmbH, Oberkochen, Germany), at an accelerating voltage of 5 kV, with an aperture size of 120 μm, a working distance of 8.4 mm, and a magnification of 5000×. It can be seen that the structural features of the composites are α phases and TiB reinforcements uniformly dispersed in the β matrix. The size of the α phase is obviously smaller than that of TiB. Precipitation of the fine α phases and the α/β interfaces can effectively impede dislocation motion, thereby enhancing the strength and toughness of the matrix [12]. TiB phase exhibits a whisker-like morphology because the growth rate along the [010] direction is faster when TiB grows in a titanium matrix, regardless of whether it is in α-Ti or β-Ti [13,14]. In addition, numerous α-Ti/TiB interfaces can be observed, which is attributed to the fact that TiB acts as a low-energy nucleation site for α-Ti [15].

**Figure 2:**
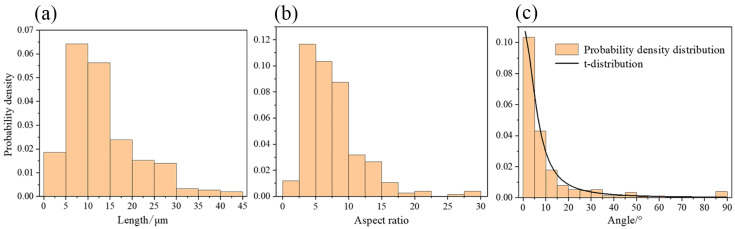
Statistical distributions of TiB particles: (**a**) length; (**b**) aspect ratio; (**c**) orientation angle.

The length, aspect ratio and orientation of TiB particles were statistically analyzed, and the results are shown in Figure 2a, Figure 2b and Figure 2c, respectively. The average length is 13.34 μm, and the aspect ratio is 7.60. TiB whiskers showed a distinct alignment along the forging direction as a result of forging. The orientation angle distribution was fitted with a location-scale t-distribution.

### 2.2. Tensile Test and Fractography

**Figure 3:**
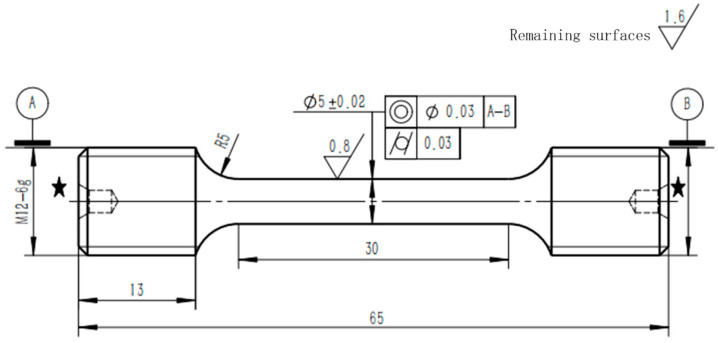
Dimensions of the tensile specimen.

Tensile tests at room temperature and SEM (Scanning Electron Microscope) observations of the tensile fracture surfaces were conducted on the TiB/Ti55531 composite. The tensile tests were performed based on the GB/T 228.1-2021 standard [16] using a universal testing machine (WDW3020, Xinke, Changchun, China) at NCS Testing Technology Co., Ltd. (Beijing, China). The tensile rate was 0.0025 s^−1^ under strain control before yielding and 0.0067 s^−1^ under displacement control after yielding. The dimensions of the tensile specimen are shown in Figure 3. SEM observations were carried out using the same equipment as described in Section 2.1.

**Figure 4:**
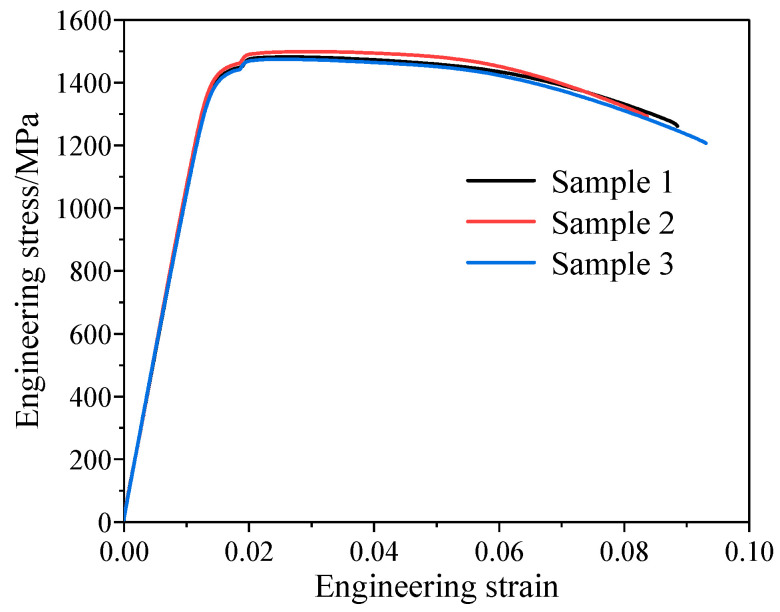
Tensile engineering stress-strain curve of the TiB/Ti55531 composite.

**Figure 5:**
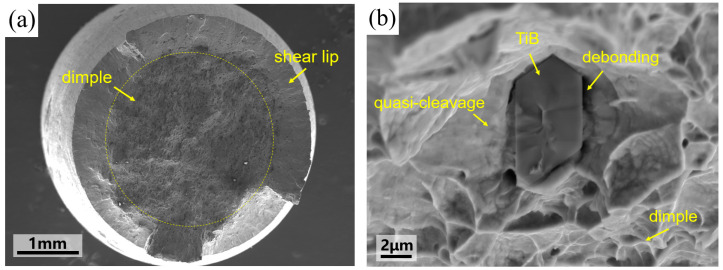
Tensile fracture surface of the TiB/Ti55531 composite: (**a**) overall view; (**b**) local view.

The engineering stress–strain curve of the TiB/Ti55531 composite obtained from the tensile test is shown in Figure 4. It can be seen that the composites show a long damage process without an obvious hardening stage. The tensile fracture surface examined by SEM is shown in Figure 5. SEM observations were conducted at an accelerating voltage of 10 kV with an aperture size of 120 μm. The working distances for Figure 5a,b were 23.2 mm and 9.5 mm, with corresponding magnifications of 18× and 5000×, respectively. It can be seen in Figure 5a that the tensile fracture surface can be divided into two main regions. The smooth ring-shaped region at the edge is the shear lip region. Shear lips usually form at the final stage of tensile fracture. The fracture direction is approximately 45° to the loading direction, which corresponds to the plane of maximum shear stress. The interior region occupies most of the fracture surface and shows typical fibrous features. Numerous dimples are observed in this region, indicating that the matrix fails mainly by ductile fracture. Fractured particle residues can be found in Figure 5b, with little interfacial debonding at the TiB particle fracture surface. In addition, the traces of plastic constraint can be observed in the matrix surrounding the particles, indicating that the interface between TiB and Ti55531 is well bonded and the reinforcement phase can effectively transfer the applied load. Under the strong interfacial constraint, the matrix near the TiB particles shows local quasi-cleavage features, while the matrix away from the particles mainly exhibits dimpled ductile fracture. Overall, the TiB/Ti55531 composite is dominated by ductile fracture of the matrix and brittle fracture of the particles, with local quasi-cleavage occurring around TiB particles and little interfacial debonding between the particles and matrix.

## 3. Microscale Interface Modeling

### 3.1. Potential Function

Potential functions determine the interactions between atoms. Therefore, selecting an appropriate interatomic potential is crucial for conducting accurate MD simulations [17]. In this study, the Second-Nearest Neighbor Modified Embedded Atom Method (2NN-MEAM) potential developed by Attarian et al. [18] was adopted. This potential has been successfully applied to the Ti-TiB system. The form of the 2NN-MEAM potential is shown in Equation (1).

(1) $$E=\sum_{i}F\left({\bar{\rho }}_{i}\right)+\frac{1}{2}\sum_{i}\sum_{j\neq i}\Phi \left(R_{ij}\right)$$

In Equation (1), the first term represents the embedding energy, where F is the embedding function and $\rho_{i}$ is the local background electron density contributed by all other atoms except atom *i* at their respective positions. The second term denotes the conventional pair interaction potential, where $\Phi \left(R_{ij}\right)$ is the pair interaction between atoms *i* and *j* by a distance $R_{ij}$.

### 3.2. MD Modeling

**Figure 6:**
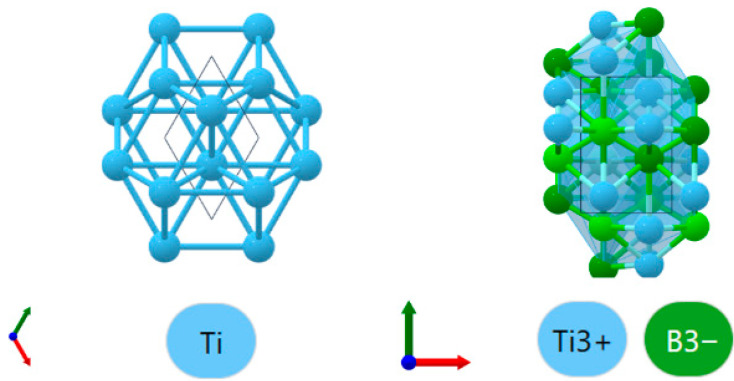
Lattice models.

According to the typical orientation relationship of the interface ${\left(0001\right)}_{\alpha -Ti}//{\left(001\right)}_{TiB},{\left[11\bar{2}0\right]}_{\alpha -Ti}//{\left[010\right]}_{TiB}$ [19], a Ti/TiB interface model at the microscale was constructed by Material Studio. Lattice models of α-Ti and TiB were obtained from the website Materials Project, as shown in Figure 6. α-Ti phase has a hexagonal crystal structure (a = 2.93573 Å, c = 4.64086 Å), while TiB has an orthorhombic structure (a = 6.10636 Å, b = 3.05025 Å, c = 4.55887 Å).

**Figure 7:**
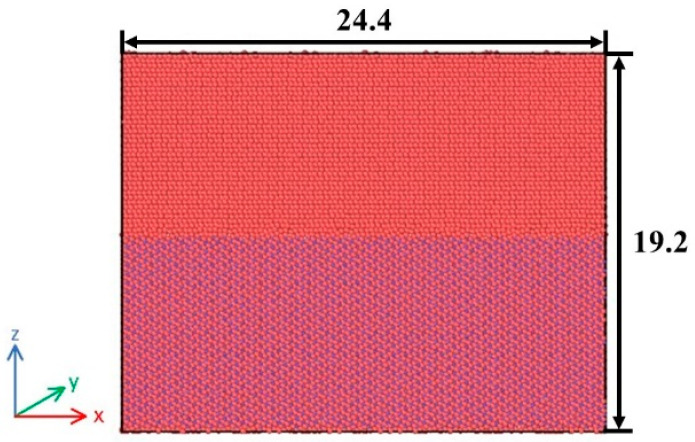
Microscale interface model (the unit is nm).

The supercell model was generated by extending unit cells along the planes (0001)_α-Ti_ and (001)_TiB,_ respectively. They were then combined to construct a microscale interface model, where x, y and z coordinate axes correspond to the crystallographic directions [010], [100] and [001], respectively. This model has dimensions of 24.4 nm × 12.0 nm × 19.2 nm and contains a total of 42,400 atoms. This size allows the interface region to contain enough atoms while keeping the computational cost reasonable, so that the results are statistically representative. At the same time, the thickness of 19.2 nm in the z direction ensures that both phases have sufficient bulk regions along the interface normal. MD simulations were performed using Lammps (2 August 2023) [20], and the results were processed and analyzed with visualization software named Ovito (3.10.6) [21]. To obtain a stable interfacial configuration with the lowest energy before loading, the model was first subjected to energy minimization using the conjugate gradient (CG) algorithm. Then, relaxation was carried out for 50 ps under the NPT ensemble at a temperature of 300 K and a pressure of 0, with periodic boundary conditions applied in all three directions. The NPT ensemble allows the system pressure to relax to zero before loading, providing a stable initial configuration. Temperature was controlled using the Nose-Hoover thermostat [22,23]. The Nose-Hoover thermostat is one of the most widely used temperature control methods in MD simulations. It introduces an equivalent heat-bath variable to regulate energy exchange and maintain a stable temperature, while preserving the realistic dynamical behavior of the system. The relaxed model is shown in Figure 7, where the upper part is Ti and the lower part is TiB.

To simulate the tensile and shear processes of the interface, a 3 nm region at the bottom of the model was fixed, while a uniform velocity of 0.5 Å/ps was applied to the top 3 nm region along the loading direction. The system was loaded under the NVT ensemble, maintaining the temperature of the free-moving layer at 300 K. The NVT ensemble allows the mechanical response to be obtained without influence from volume regulation. In addition to the constant velocity applied to the driving layer, a uniform deformation velocity gradient was imposed on the free-moving layer in the initial conditions to avoid the generation of shock waves during simulation [24]. To eliminate the influence of atomic motion caused by tension or shear on temperature calculation, the temperature of the free-moving layer was computed using atomic velocities only in the two directions perpendicular to the loading direction. For tensile simulation, periodic boundary conditions were applied in the x and y directions, while the z direction was non-periodic. For shear simulation, periodic boundary conditions were applied in the y direction, while x and z directions were non-periodic.

### 3.3. Traction-Separation Model

To parameterize CZM, it is necessary to obtain T-S curve data from MD simulations. Atoms within 15 Å above and below the interface were selected for statistical analysis. Traction was calculated as the volume-averaged virial stress of all atoms in these regions along the loading direction [25]. Separation was determined by subtracting the average displacement of atoms in a 15 Å region below the interface from that of the 15 Å region above the interface along the loading direction. Based on the T-S responses obtained from the MD simulations, a bilinear fitting was performed to extract the interfacial stiffness, peak strength and fracture energy. A parameterized CZM was then established to describe the interfacial mechanical behavior under normal and tangential loading. The obtained interfacial parameters were then introduced into the RVE model to achieve the multiscale linkage between microscale and mesoscale.

## 4. Mesoscale Representative Volume Element Modeling

### 4.1. Model Generation

**Figure 8:**
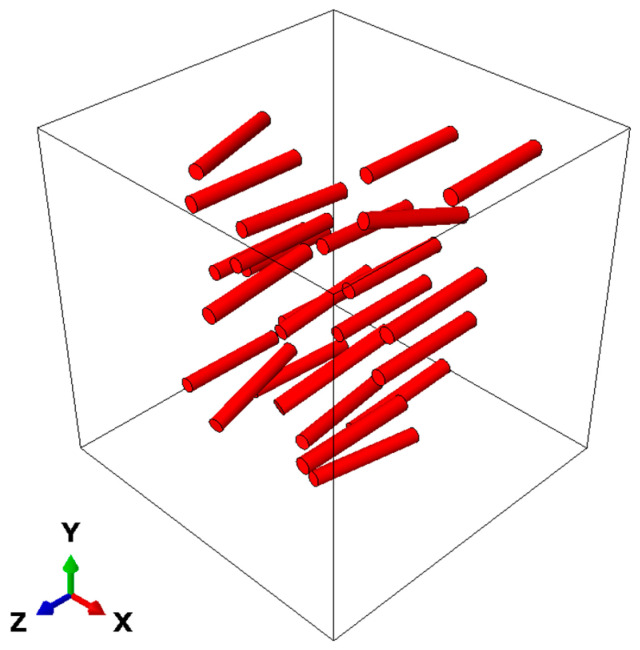
RVE model.

A three-dimensional RVE [26,27,28] model of the composite was constructed using the random sequential adsorption (RSA) algorithm [29,30]. The principle of RSA is to randomly generate particles in a cubic space and determine whether each newly generated particle intersects with any previously placed ones. If no intersection occurs, the particle is retained; otherwise, a new particle is regenerated until the preset volume fraction is reached. TiB reinforcements were modeled as cylinders. Geometric parameters of the generated particles were determined based on experimental statistical results presented in Section 2.1. Length and aspect ratio were taken as average values from statistics. Orientation was randomly generated using the t-distribution fitted in Figure 2a as the probability density function. The particle volume fraction was 1.25%, matching the experimental material. The constructed model is shown in Figure 8, and the size of the cube is 40 μm. To avoid distorted elements during finite element meshing, the generated particles were prevented from being too close to each other or to the boundaries of the cube.

### 4.2. Materials Constitutive

For the Ti55531 matrix, isotropic hardening is described as [31]:

(2) $$\sigma =\sigma_{y}{\left(1+\frac{E}{\sigma_{y}}\varepsilon_{p}\right)}^{n}$$

where σ is the current stress, $\sigma_{y}$ is the yield strength, E is the Young’s modulus, $\varepsilon_{p}$ is the plastic strain, and n is the hardening exponent.

Except for the direct strengthening provided by the load transfer effect of TiB reinforcements, TiB also causes indirect strengthening by modifying the microstructure or the state of the Ti55531 matrix. Indirect strengthening mainly includes matrix grain refinement and thermal mismatch strengthening, etc. For TiB/Ti55531 composites, previous studies have shown that the addition of reinforcements leads to significant grain refinement of the matrix [19]. Since the difference in thermal expansion coefficients between TiB and Ti is relatively small (Ti = 8.2 × 10^−6^/°C and TiB = 7.2 × 10^−6^/°C) [5], thermal mismatch strengthening can be neglected. Lu et al. [32] also reported that in similar materials (TiC + TiB reinforced titanium matrix composites), the strengthening effect mainly arises from the direct load-bearing of particles and the grain refinement of the matrix. Therefore, the indirect strengthening mechanism is primarily attributed to the grain refinement effect. The increase in yield strength induced by grain refinement can be expressed by the Hall-Petch relationship [33,34]:

(3) $$∆\sigma_{gb}=KD_{HP}^{-\frac{1}{2}}$$

(4) $$D_{HP}=d_{HP}{\left[\left(1-F_{V}\right)/F_{V}\right]}^{\frac{1}{3}}$$

where K is the Hall-Petch slope, $D_{HP}$ is the equivalent grain size, $d_{HP}$ is the particle size, and $F_{V}$ is the particle volume fraction.

For ductile damage fracture of Ti55531, the Rice and Tracey criterion is adopted to evaluate the damage level [35]:

(5) $$D_{\eta }=\int_{0}^{{\bar{\varepsilon }}^{pl}}e^{\frac{3}{2}\eta }d{\bar{\varepsilon }}^{pl}$$

(6) $${\bar{\varepsilon }}^{pl}=\int_{0}^{t}{\dot{\bar{\varepsilon }}}^{pl}dt$$

(7) $${\dot{\bar{\varepsilon }}}^{pl}=\sqrt{\frac{2}{3}\dot{\varepsilon }:\dot{\varepsilon }}$$

where $D_{\eta }$ is the damage parameter in the Rice and Tracey criterion. When it reaches $D_{\eta c}$, the matrix element begins to fail. η, ${\bar{\varepsilon }}^{pl}$, ${\dot{\bar{\varepsilon }}}^{pl}$ and $\dot{\varepsilon }$ correspond to the stress triaxiality, equivalent plastic strain, equivalent plastic strain rate and plastic strain rate tensor, respectively.

The post failure behavior plays a significant role in predicting the stress–strain response after damage initiation in RVE. This process can be simulated by reducing the stiffness of the elements. In this study, a linear damage evolution law was adopted, which is defined as [35]:

(8) $$d=min\left(1,k_{d}\frac{\varepsilon_{eq}-\varepsilon_{0}}{\varepsilon_{0}}\right)$$

where d is the damage factor, and the element completely fails when d=1. $\varepsilon_{eq}$ and $\varepsilon_{0}$ represent the equivalent strains at the current state and at the onset of failure, respectively. $k_{d}$ is an adjustment coefficient. The above criterion was implemented through a VUMAT subroutine in Abaqus. For TiB, brittle fracture was implemented through the “brittle cracking model” in Abaqus.

Material parameters of Ti55531 and TiB are listed in Table 1 [36,37,38,39].

**Figure 9:**
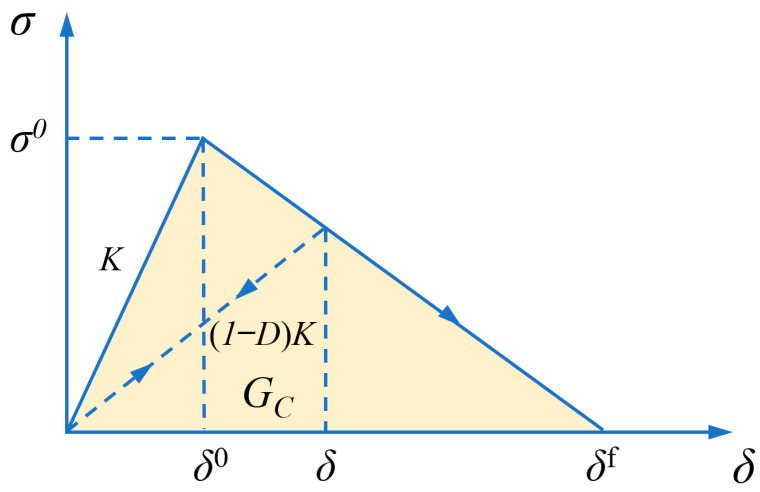
The traction-separation behavior of bilinear CZM.

A CZM was adopted for interfacial damage [40]. CZM defines interfacial behavior through a traction-separation relationship, in which the matrix and reinforcement transfer traction across the interface. When the traction exceeds the maximum allowable value, the interfacial stiffness gradually decreases until it can no longer carry any load, leading to debonding. The typical shape of the T-S curve is bilinear, as shown in Figure 9, where *G_C_* represents the interfacial fracture energy.

The bilinear T-S curve mainly includes three criteria [41,42]:

(1) Traction-separation criterion

Before damage occurs, the stress and separation displacement on the interface follow a linear elastic relationship:

(9) $$t=\left\{\begin{array}{c} t_{n}\\ t_{s}\\ t_{t} \end{array}\right\}=\left[\begin{array}{ccc} K_{n} & 0 & 0\\ 0 & K_{s} & 0\\ 0 & 0 & K_{t} \end{array}\right]\left\{\begin{array}{c} \delta_{n}\\ \delta_{s}\\ \delta_{t} \end{array}\right\}=K\delta$$

where t is the traction stress vector, $t_{n}$, $t_{s}$ and $t_{t}$ are the nominal stress components in the normal and two orthogonal tangential directions, respectively. K is the interfacial elastic stiffness matrix, δ is the separation displacement vector, and $\delta_{n}$, $\delta_{s}$, $\delta_{t}$ are the separation components in the normal and two tangential directions.

(2) Damage initiation criterion

Damage begins to occur when the condition of the damage initiation criterion is satisfied. The quadratic yield criterion is used to assess the damage initiation of CZM, which is described as

(10) $${\left\{\frac{t_{n}}{t_{n}^{0}}\right\}}^{2}+{\left\{\frac{t_{s}}{t_{s}^{0}}\right\}}^{2}+{\left\{\frac{t_{t}}{t_{t}^{0}}\right\}}^{2}=1$$

where $t_{n}^{0}$, $t_{s}^{0}$ and $t_{t}^{0}$ correspond to the maximum nominal stresses for pure Mode I, Mode II and Mode III failures, respectively.

(3) Damage evolution criterion

After the initiation of damage, linear degradation of the interface stiffness occurs until complete failure. The damage variable D is defined as

(11) $$D=\frac{\delta^{f}\left(\delta -\delta^{0}\right)}{\delta \left(\delta^{f}-\delta^{0}\right)}$$

where $\delta^{0}$, δ and $\delta^{f}$ correspond to the equivalent interfacial separation at damage initiation, current equivalent interfacial separation and maximum equivalent interfacial separation.

### 4.3. Simulation Settings

The tensile simulation of the RVE was carried out using Abaqus/Explicit. To ensure computational accuracy, the mesh type C3D10M was adopted for both Ti matrix and TiB reinforcements, while the interfacial damage model was implemented through a contact-based CZM in Abaqus.

**Figure 10:**
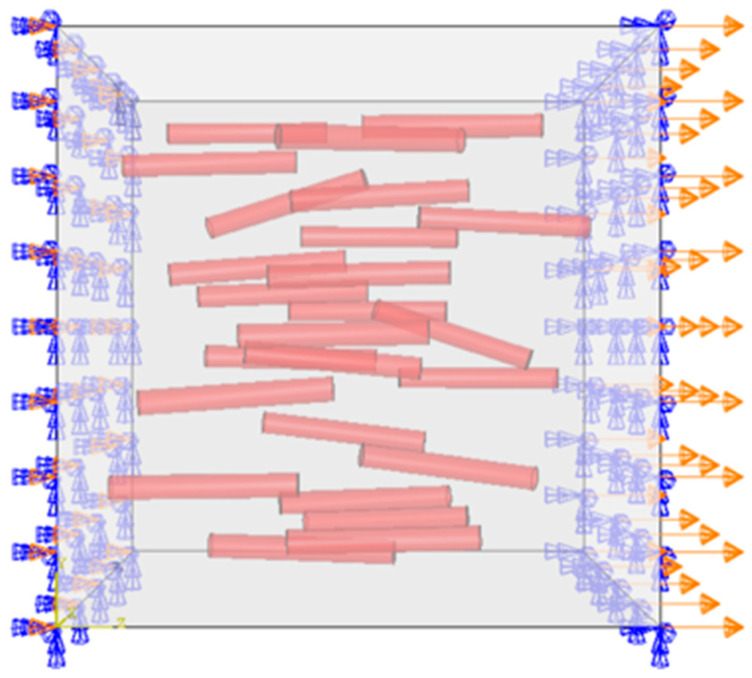
Schematic image of loading and boundary conditions on the RVE.

Although the periodic boundary condition (PBC) can provide accurate results under certain circumstances, it introduces intense high-frequency oscillations in the system, which compromise the numerical solutions [43]. Moreover, simulations using PBC are very computationally expensive and are not suitable for fracture modeling. In this study, a displacement loading was applied by fixing the displacement of the Y-Z plane in the X direction and imposing a uniaxial displacement on the parallel Y-Z plane in the X direction. Furthermore, to guarantee model stability after fracture, the rotational degrees of freedom of both the fixed and loading faces were constrained in all three directions [44]. The applied boundary conditions are illustrated in Figure 10.

In finite element analysis, the homogenized nominal stress and strain are typically calculated using the following formulas:

(12) $$\sigma_{nom}=\frac{\sum_{1}^{N}f_{RF}}{L^{2}}$$

(13) $$\varepsilon_{nom}=\frac{\Delta x}{L}$$

In the equations, $f_{RF}$ is the reaction force of nodes on the loading surface, L is the initial edge length of the RVE, and Δx is the displacement along the loading direction.

**Figure 11:**
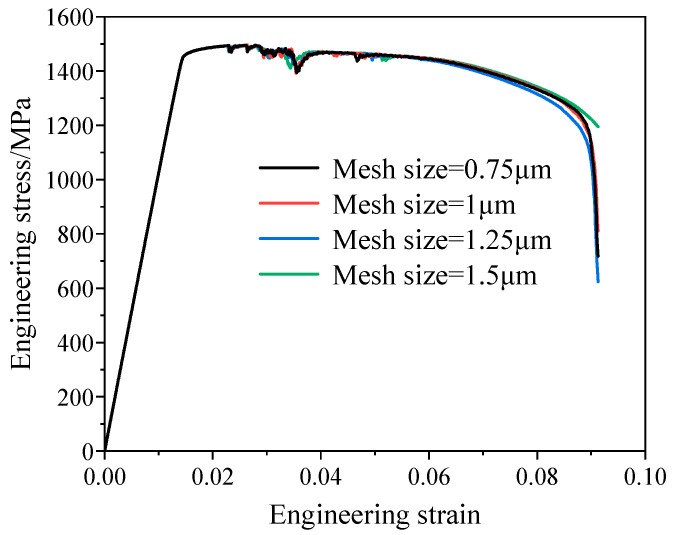
Effect of mesh size on simulation results.

In finite element simulations, the choice of mesh parameters plays a significant role in the accuracy of numerical results. In this work, the convergence of the numerical simulation results with respect to mesh size was analyzed. The RVE was subjected to tensile simulations using different mesh sizes of 0.75, 1.0, 1.25, and 1.5 μm, and the results were compared, as shown in Figure 11. Among them, the simulated curves obtained with mesh sizes of 0.75 μm and 1.0 μm almost completely overlap, indicating that the results tend to be stable, while deviations are observed for mesh sizes of 1.25 μm and 1.5 μm. Considering both computational accuracy and efficiency, a mesh size of 1.0 μm was selected, which corresponds to 814,019 elements.

## 5. Results and Discussion

### 5.1. Microscale Interfacial MD Simulations

**Figure 12:**
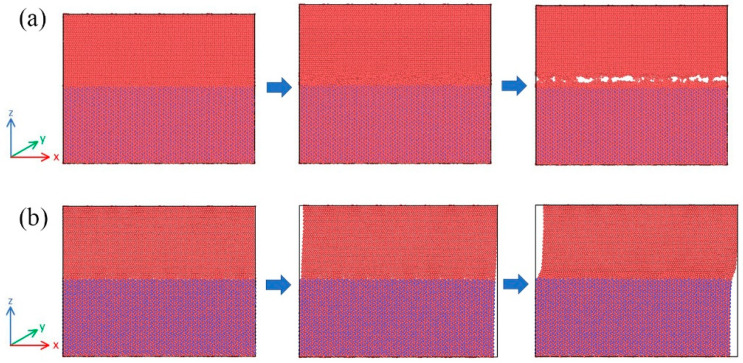
Interfacial behavior obtained from MD simulations: (**a**) tension; (**b**) shear.

The tensile and shear simulation results of the interfacial behavior are shown in Figure 12a,b. During tensile deformation, the model is first stretched, and the traction increases rapidly with increasing separation until it reaches a peak. Meanwhile, the atomic arrangement of the Ti matrix near the interface gradually becomes disordered. Subsequently, numerous tearing voids appear in the Ti matrix near the interface, which soon lead to a complete Mode I fracture. At the same time, the traction drops rapidly and eventually reduces to zero. During shear deformation, the TiB region exhibits almost no deformation because its stiffness is much higher than that of Ti. The Ti matrix undergoes an overall shear deformation, and the traction rises quickly with increasing separation until reaching a peak. As the simulation proceeds, shear deformation becomes concentrated in the Ti matrix near the interface. At this stage, the Ti matrix begins to degrade and the traction decreases. By comparing the tensile and shear simulations, it can be seen that in the tensile simulation, there is a clear fracture moment, while the interfacial damage in the shear simulation progresses gradually.

**Figure 13:**
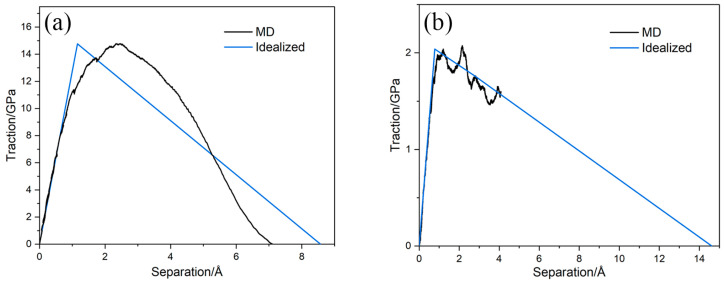
T-S responses with fitted curves: (**a**) tension; (**b**) shear.

Figure 13a,b, respectively, display the tensile and shear T-S responses along with their bilinear fitted curves. For the tensile curve, the first line in the bilinear curve is drawn using the slope of the actual curve at the initial stages of deformation and is extended up to the peak stress. The second line is drawn such that the area under the bilinear graph is equal to the area under the actual curve obtained from the MD simulation. The interfacial normal stiffness is 1.276 × 10^11^ MPa/mm, peak stress is 14.76 GPa, and fracture energy is 6.33 J·m^−2^ from the fitted results. For the shear curve, the first line in the bilinear curve is drawn based on the initial slope of the actual curve and is extended up to the maximum shear stress. Then, for the second line, the second peak of the actual curve is used, and a linear degradation of the shear strength is assumed. For the other shear direction, the same fitting relationship is assumed. The interfacial tangential stiffness is 2.611 × 10^10^ MPa/mm, peak tangential stress is 2.04 GPa, and fracture energy is 1.49 J·m^−2^ from the fitted results. The normal interfacial stiffness, peak stress, and fracture energy under tensile deformation are significantly higher than the tangential ones, indicating that shear failure is more likely to occur. The obtained interfacial parameters are used as the CZM constitutive parameters and then introduced into the mesoscale RVE simulations to characterize interfacial behavior.

### 5.2. Mesoscale RVE Simulations

**Figure 14:**
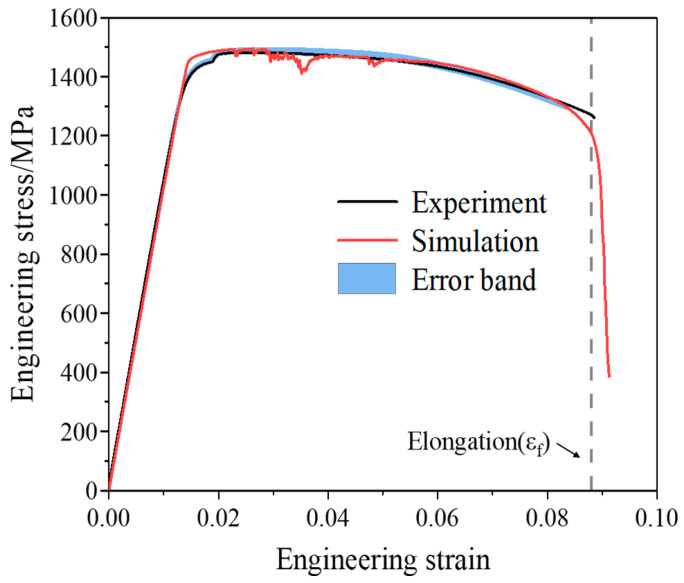
Comparison of the experimental and simulated tensile engineering stress–strain curves.

Figure 14 shows the engineering stress–strain curve obtained from the mesoscale RVE simulation as well as the experimental curve. The mechanical property parameters extracted from the curves are listed in Table 2. Although the mesoscale RVE model is still different in scale from the actual experimental specimen, many studies have shown that such a comparison is effective and common [11,31,35,44]. This is due to the fact that, when the RVE is large enough and contains the main statistical features of the real microstructure, it can represent the average mechanical behavior and thus be used to predict macroscopic properties. It can be seen that the experimental and simulated curves agree well. They almost overlap in the elastic stage, while some oscillations appear in the simulation during the plastic and damage-evolution stages due to particle fracture. In addition, a certain deviation from the experimental curve is observed in the final failure stage. It indicates that the RVE still has certain limitations. Due to its boundary conditions and geometric constraints, the RVE may not fully capture the stress redistribution and geometric nonlinearity that occur in the later stages of fracture, especially after necking begins. Therefore, some differences may appear between the RVE predictions and the experimental results in the final failure stage. Nevertheless, the overall agreement with the experimental result remains remarkably high. The elastic modulus, yield strength, ultimate tensile strength and elongation show only small differences from the experimental values, with the maximum error not exceeding 3.6%. It is noted that the elongation in the simulation is determined by dividing the tensile process into 100 equal steps. Each step corresponds to a stress value $\sigma_{i}$, and the strain at $\sigma_{i}$ is taken as the elongation when the following condition is satisfied:

(14) $$\frac{\sigma_{i+1}-\sigma_{i}}{\sigma_{i}}<-5\%$$

**Figure 15:**
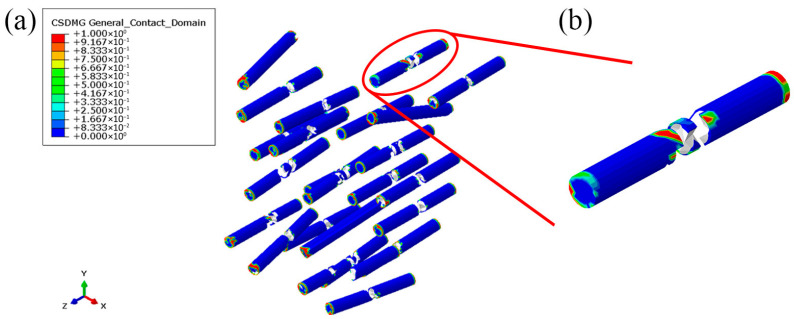
Simulated particle fracture and debonding: (**a**) overall view; (**b**) local view.

Figure 15 shows the simulated particle fracture and debonding, where the red color represents complete debonding. It can be seen that most particles are fractured from the middle. In addition, partial interface debonding appears at the fracture surfaces and at the ends of the particles. This is consistent with the observations from the experiment and the references. Specifically, the in situ TiB/Ti interface is strong, where the particles can effectively carry the applied load and thus fracture. In addition, interfacial debonding is limited and usually occurs at the fracture surfaces or at the particle ends. This indicates that the interfacial parameters obtained from the MD simulations are reasonable. Meanwhile, the simulation matches the experimental results very well in terms of the tensile curves, mechanical property parameters and fracture modes, which confirms the rationality and effectiveness of the mesoscale RVE model. This indicates that the model can be used to further investigate the damage process and the effects of various factors on the material’s mechanical behavior.

## 6. Conclusions

In this paper, a multiscale simulation method from microscale to mesoscale for investigating the mechanical properties of in situ particle reinforced TiB/Ti55531 composites is proposed. The mechanical properties of the composites, which are analyzed by the representative volume element (RVE), show excellent agreement with the experimental results. Using the proposed multiscale finite element model, several important findings for in situ particle reinforced TiB/Ti55531 composites are summarized:

1. A microscale TiB/Ti interface model is established and analyzed by molecular dynamics (MD) simulation using the 2NN-MEAM potential. The fundamental traction–separation (T–S) relation of the interface was obtained from the MD results. The simulation results indicate that the normal interfacial fracture energy and the tangential fracture energy at 300 K are 6.33 J·m^−2^ and 1.49 J·m^−2^, respectively.
2. In the mesoscale, an RVE model is generated in accordance with the microstructural observation of the titanium matrix composite. The cohesive zone model (CZM) is introduced into the finite element simulation to describe the interfacial behavior. Furthermore, the ductile damage of the matrix and brittle fracture of the particles are considered to determine failure.
3. The simulated results, including tensile stress–strain curve, Young’s modulus, tensile strength and elongation, show excellent agreement with the experiments. Meanwhile, the particle fracture and debonding behaviors are also simulated in the multiscale model. It shows that most particles are fractured from the middle, and some interface debonding appears at the ends of the particles. This is also consistent with the fracture morphology observation of the samples.

## Figures and Tables

**Table 1:** Material parameters.

Properties	Symbol	Unit	Ti	TiB
Density	*ρ*	g/cm^3^	4.635	4.5
Young’s modulus	*E*	GPa	100.11	450
Poisson’s ratio	*ν*	/	0.285	0.166
Yield/Fracture stress	*σ*	MPa	1365	8000

**Table 2:** Experimental and simulated mechanical properties.

Properties	Symbol	Unit	Exp	Sim	Error
Young’s modulus	*E*	GPa	106.36	103.53	−2.7%
Yield stress	*σ*_0.2_	MPa	1420.44	1472.36	3.6%
Tensile stress	*σ_max_*	MPa	1485.13	1496.54	0.8%
Elongation	*ε_f_*	%	8.84	8.8	−0.5%

## Data Availability

The original contributions presented in this study are included in the article. Further inquiries can be directed to the corresponding author.
